# Dihydroxy-Substituted Coumarins as Fluorescent Probes for Nanomolar-Level Detection of the 4-Amino-TEMPO Spin Label

**DOI:** 10.3390/ijms20153802

**Published:** 2019-08-03

**Authors:** Krzysztof Żamojć, Magdalena Zdrowowicz, Aleksandra Hać, Maciej Witwicki, Paweł Błażej Rudnicki-Velasquez, Dariusz Wyrzykowski, Wiesław Wiczk, Lech Chmurzyński

**Affiliations:** 1 Faculty of Chemistry, University of Gdańsk, Wita Stwosza 63, 80-308 Gdańsk, Poland; 2 Faculty of Biology, University of Gdańsk, Wita Stwosza 59, 80-308 Gdańsk, Poland; 3 Faculty of Chemistry, University of Wrocław, F. Joliot-Curie 14, 50-383 Wrocław, Poland

**Keywords:** nitroxide radicals, dihydroxycoumarins, fluorescence, optical chemical sensors

## Abstract

This paper reports on dihydroxycoumarins as fluorescent probes suitable for the detection and determination of the nitroxide radical, namely 4-amino-TEMPO. Since 4-amino-TEMPO is used as a spin label for the detection of various radicals and damage caused by these species, its determination under physiological conditions might help us to understand the mechanism of the oxidative stress. Among different coumarins studied, only dihydroxy-substituted derivatives show high sensitivity, specificity, and selectivity for the nitroxide radical. In this assay, dihydroxy-substituted coumarins under the action of 4-amino-TEMPO show a very fast and significant increase in fluorescence intensity and lifetime. Among them 6,7-dihydroxycoumarin (esculetin) exhibits the strongest fluorescence enhancement (up to 40 times), with an estimated limit of detection equal to 16.7 nM—a significantly lower value when compared with UV-Vis or electron paramagnetic resonance (EPR) spectroscopy. The method is characterized by an easy procedure of sample preparation and very short time of analysis. The mechanism of the interaction between 6,7-dihydroxycoumarin and 4-amino-TEMPO has been examined with the use of a series of complementary techniques, such as steady-state and time-resolved fluorescence spectroscopy, UV-Vis spectroscopy, electron paramagnetic resonance spectroscopy, potentiometric titration, and high-performance liquid chromatography. It has been proven that the only route of the reaction in the system studied is a proton transfer from the molecule of esculetin to the amino group of the nitroxide. Biological studies performed on prostate cancer cells, breast cancer cells, and normal skin fibroblasts revealed significant anticancer properties of 6,7-dihydroxycoumarin, which caused a considerable decrease in the viability and number of cancer cells, and affected their morphology, contrary to normal fibroblasts. Furthermore, the experiment performed on prostate cancer cells showed that fluorescence emission of esculetin is closely related to intracellular pH—the higher pH, the higher observed fluorescence intensity (in accordance with a chemical experiment). On the other hand, the studies performed in different pH levels revealed that when pH of the solution increases, the observed fluorescence intensity enhancement under the action of 4-amino-TEMPO decreases (better sensing properties of esculetin towards the nitroxide in lower pH).

## 1. Introduction

Reactive oxygen and nitrogen species (RONS), e.g., superoxide, hydroxyl radical, singlet oxygen, nitric oxide, nitrogen dioxide, peroxynitrite, and hydrogen peroxide, are powerful oxidants that may damage cellular targets non-selectively [1]. Radicals, including RONS, represent a broad range of short-lived and chemically distinct individuals [2]. Therefore, they are very difficult to detect in dynamic environments such as biological media, where the presence of a variety of endogenous antioxidants additionally complicates their determination [3].

One of the methods suitable for the quantitative measurement of an oxidative stress status—based on a determination of the total content of radicals—involves the use of exogenous nitroxides as probes of the red-ox balance in each environment. Nitroxide radicals based on 2,2,6,6-tetramethylpiperidinyl-*N*-oxyl (TEMPO) are highly stable, isolable species. Although the addition of TEMPO to alkyl and acyl radicals occurs below the diffusion-controlled limit, these reactions proceed more readily than dimerization or other self-reactions of the nitroxide [4]. A variety of TEMPO derivatives are therefore used as spin labels for supramolecular complexes [5]; reversible inhibitors for nitroxide-mediated polymerization [6]; probes for the detection of various radicals and the damage mediated by these species [7,8,9,10,11,12]; prefluorescent probes [13,14]; and as superoxide dismutase mimetics for the protection of biomolecules against oxidative stress [15,16]. Similarly, derivatives of TEMPO can act as radical scavenging, anti-oxidant stabilizers for polymers, improving durability and aesthetic properties throughout their service lifetime [4].

4-Amino-TEMPO itself is known to possess superoxide dismutase-mimetic activity in vitro, penetrate easily the cells and protect them against oxidative damage caused by H_2_O_2_ [17]. It has been proven that 4-amino-TEMPO exhibits significant radioprotective properties and—due to its ability to maintain the positive charge—protects DNA effectively from damages induced by oxidative stress generated during UV light exposure [18]. Quantum dot-4-amino-TEMPO complexes have been reported as highly selective prefluorescent sensors for the detection of radicals, since the fluorescence of the quantum dots, which is quenched by the nitroxide, is readily restored when carbon-centred radicals are combined with the nitroxide moiety to form diamagnetic alkoxyamines [19,20]. Furthermore, 4-amino-TEMPO and other derivatives of TEMPO are used in a variety of industrial applications as highly selective oxidation catalysts to produce pharmaceuticals, flavours and fragrances, agrochemicals, and a variety of other specialty chemicals [21]. Therefore, the development of highly efficient methods for the selective detection and determination of 4-amino-TEMPO radical is highly desirable. This information may be very important both in clarifying the mechanism of polymer stabilization as well as in the technological search for better additives (or their mixtures) exhibiting a synergetic protective action for polymers [22].

Although the level of the nitroxide radicals can be evaluated by electron paramagnetic resonance (EPR) spectroscopy, spectrofluorimetry seems to be a great alternative, namely due to its simplicity, high sensitivity, precise quantitative determination, and real-time detection [23]. Moreover, the approach of the fluorescence imaging is the best technique for the detection and quantitation of intracellular molecules without the destruction of tissues or cells hence making the fluorescence spectroscopy superior to other analytical techniques [24]. Although many fluorescent probes for the detection of radicals [25,26] and other RONS [27,28,29] have been already developed, there is not much information about fluorescent sensors used for the detection of nitroxides [30].

In this work we demonstrate a method for the detection and determination of 4-amino-TEMPO in aqueous solution using dihydroxycoumarins as fluorescent probes. We have previously examined the interactions between another stable nitroxide radical, namely 4-hydroxy-TEMPO, and various fluorescent species, i.e., polycyclic aromatic hydrocarbons [31] and coumarins [32,33], as well as fluoroquinolone antibiotics [34]. In most cases, we observed the strictly physical interactions between the reactants and the nitroxide, which acted as a fluorescence quencher. We herein report that coumarin derivatives containing two hydroxyl substituents selectively and rapidly interact with 4-amino-TEMPO; this is accompanied by significant fluorescence intensity and lifetime enhancement.

Taking into account the fact that intracellular accumulation of various lipophilic anticancer agents is modulated by the cellular pH gradient [35], considering selected hydroxy-substituted coumarins as potential chemotherapeutic drugs it is essential to discuss the intracellular and extracellular pH (pH_i_ and pH_e_, respectively) of different cancer cells. Although it has been shown that pH_i_ of a number of various multidrug resistant cell lines is higher when compared with their parental counterparts [36], generally intracellular pH is similar in both solid tumour and normal tissues [35]. On the other hand, extracellular pH is greater in normal tissues (pH_e_ typically ~7.4) and lower in solid tumours (median pH_e_ typically 6.5–7.0); hypoxia and high glycolytic activity, which are characteristic for solid tumours, lead to increased production and secretion of H^+^ to the extracellular space [37]. Such a pH gradient (not observed in normal tissues) formed between intra- and extracellular space very often affects the distribution and uptake of selected chemotherapeutics, which may result in physiological drug resistance [37]. Passive diffusion of drugs into cells occurs mainly when they are uncharged. Thus, the cellular uptake of drugs with acidic or basic properties depends on pH_e_, whereas their trapping in a charged form inside cells depends on pH_i_ [38].

## 2. Results and Discussion

### 2.1. Chemical Studies

**Figure 1:**
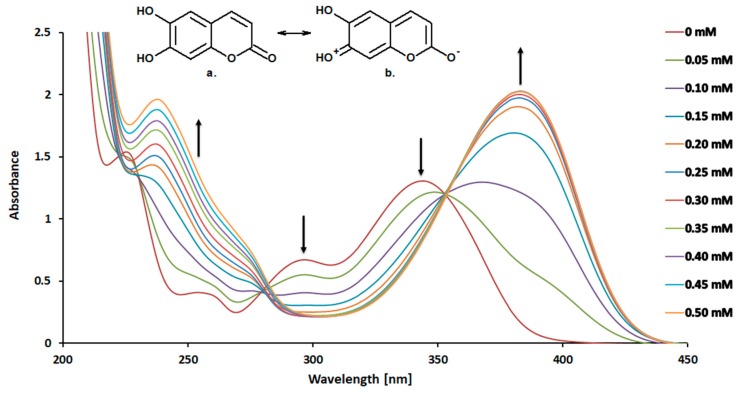
Absorption spectra of 6,7-dihydroxycoumarin (0.10 mM) in the presence of increasing concentrations (0–0.50 mM) of 4-amino-TEMPO in water at 25 °C. Inset presents the structures of two tautomers of 6,7-dihydroxycoumarin.

Figure 1 shows absorption spectra of 6,7-dihydroxycoumarin (0.10 mM) recorded in the absence and presence of increasing concentrations (up to 0.50 mM) of 4-amino-TEMPO. In the absence of 4-amino-TEMPO, the absorption spectrum of an aqueous solution of esculetin displays maxima at 298 and 345 nm. The significant variation of the spectrum of 6,7-dihydroxycoumarin from that of coumarin (the spectrum not shown) is explained by the predominant contribution of tautomer (b) to the structure of esculetin (see inset in Figure 1) [39]. At higher concentrations of 4-amino-TEMPO, the major band at 345 nm (attributed to the protonated form of the phenolic group) gradually decreases, and the peak at around 385 nm appears simultaneously with a distinct isosbestic point at 353 nm. The decline of the band at 345 nm along with arise of a new band at around 385 nm has also been noticed for all mono- and dihydroxy-substituted coumarins under study (overall 12 derivatives). It is worth mentioning that such observations were not found when these analogues were mixed with TEMPO itself and other derivatives of the nitroxide, i.e., 4-hydroxy-TEMPO, 4-methoxy-TEMPO, 4-acetamido-TEMPO, 4-oxo-TEMPO, 4-carboxy-TEMPO and 4-hydroxy TEMPO benzoate (data not shown). These findings reveal that only coumarins with hydroxyl substituent/substituents are sensitive to 4-amino-TEMPO and they simultaneously remain insensitive to other derivatives of TEMPO. Since the saturation of the double bond at the 3,4-position in the coumarin nucleus greatly reduces absorption at wavelengths longer than 300 nm, that route of the reaction may be declined. It is known that chemical composition and pH of the solvent have an important influence on the shape of the absorption spectra, as shown, for example, by the curves which have been published for scopoletin (6-methoxy-7-hydroxycoumarin) in several aqueous buffers [40]. One of the reasons for the recorded bathochromic shifts of the longest-wavelength bands in electronic absorption spectra of *ortho*-dihydroxycoumarins could be an increase of electron donation of the oxygen atom of the hydroxyl group via H-bonding. In case of monohydroxy-substituted coumarins as well as dihydroxy-substituted derivatives without neighbouring hydroxyl groups, in case of which the formation of intramolecular hydrogen bond does not occur, deprotonation may be responsible for the observed shifts—the deprotonation leads to a significant increase of HOMO energy in 7-hydroxycoumarins and thus to bathochromic band shifts in their electronic absorption spectra [41]. Since the absorption spectra of all hydroxycoumarins studied recorded in the presence of 4-amino-TEMPO (which due to the presence of amino group may act as a potential proton acceptor) exhibit such a shift, the appearance of ionized forms of these derivatives cannot be excluded and should be taken into further consideration.

**Figure 2:**
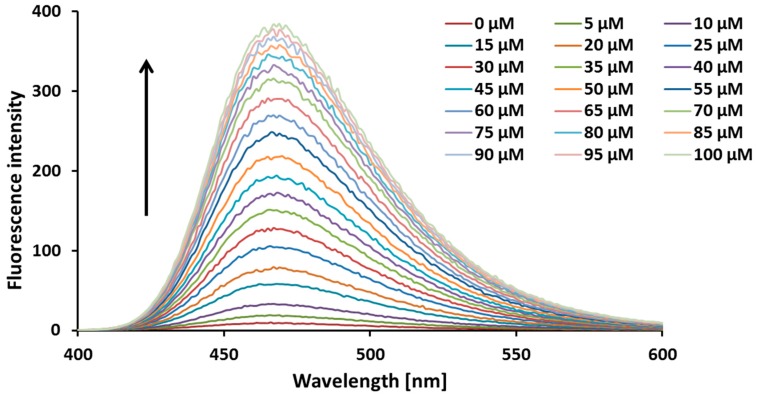
The increase in the fluorescence intensity of 6,7-dihydroxycoumarin (10 µM) with the addition of 4-amino-TEMPO (0–100 µM) in water at 25 °C.

**Figure 3:**
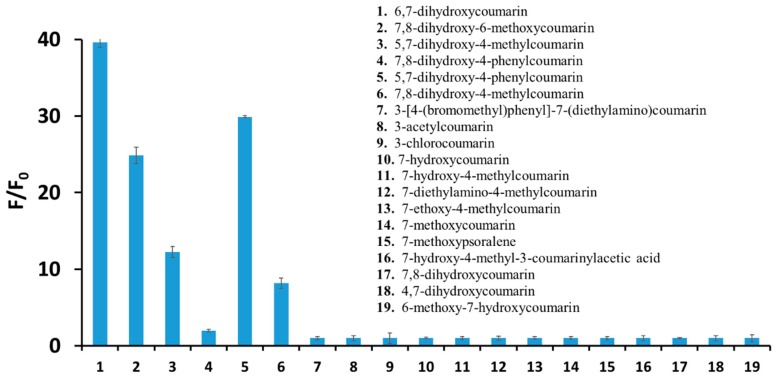
The enhancement of the fluorescence intensity of the coumarins studied (10 µM) in the presence of 4-amino-TEMPO (100 µM) in aqueous solutions. Each experiment was carried out three times, while fluorescence intensity values were the average of 15 measurements.

Figure 2 shows the fluorescence emission spectra of 6,7-dihydroxycoumarin (10 µM) registered in the absence and presence of increasing concentrations (up to 100 µM) of 4-amino-TEMPO. Upon an excitation at 350 nm, esculetin displays a fluorescence emission spectrum with the maximum at 467 nm. The addition of 4-amino-TEMPO results in a significant increase of the fluorescence intensity of esculetin. In the presence of 4-amino-TEMPO at a concentration of 100 µM, an about 40-fold fluorescence enhancement is observed. As seen in Figure 3, a significant increase in a fluorescence intensity under the action of the nitroxide is also observed for other dihydroxycoumarins studied, particularly 5,7-dihydroxy-4-phenylcoumarin (~30-fold), 7,8-dihydroxy-6-methoxycoumarin (~25-fold), and 5,7-dihydroxy-4-methylcoumarin (~13-fold). Figure S1 in Supplementary Materials shows appropriate calibration graphs reflecting the linear correlation between the relative fluorescence enhancement of these derivatives and the concentration of 4-amino-TEMPO up to 50 µM. On the other hand, in the case of coumarins with less than two hydroxyl substituents no fluorescence enhancement is observed.

**Figure 4:**
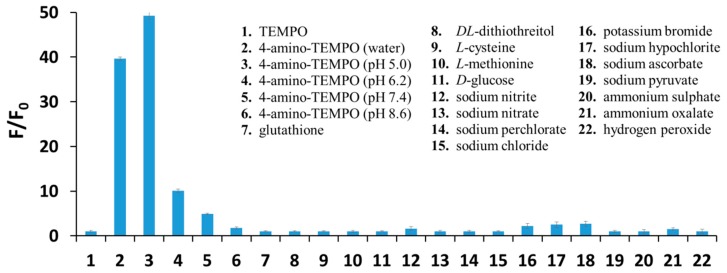
The fluorescence enhancement of 6,7-dihydroxycoumarin (10 µM) in the presence of TEMPO (negative control), 4-amino-TEMPO in different pH and various redox agents, and other species present under biological conditions (100 µM). Each experiment was carried out three times, while fluorescence intensity values were the average of 15 measurements.

To evaluate the selectivity of dihydroxycoumarins towards 4-amino-TEMPO, 6,7-dihydroxycoumarin was exposed to typical redox agents, as well as other species commonly present under biological media, i.e., glutathione, dl-dithiothreitol, l-cysteine, l-methionine, d-glucose, sodium nitrate, sodium nitrite, sodium perchlorate, sodium chloride, potassium bromide, sodium hypochlorite, sodium pyruvate, sodium ascorbate, ammonium sulphate, ammonium oxalate, histamine dihydrochloride, and hydrogen peroxide. 6,7-Dihydroxycoumarin showed either no fluorescence enhancement or a negligible increase in fluorescence under the action of these species in aqueous solutions (Figure 4). Furthermore, from the inspection of Figure 4 one can observe that the lower the pH of the solution the greater the fluorescence enhancement of 6,7-dihydroxycoumarin under the action of 4-amino-TEMPO, and thus the better the sensing properties of esculetin towards the studied nitroxide radical. Finally, it indicates that selected dihydroxycoumarins may be successfully applied as fluorescent probes for a determination of 4-amino-TEMPO in physiological pH as well, although fluorescence enhancement of 6,7-dihydroxycoumarin in the presence of 100 μM 4-amino-TEMPO is approximately 10 times smaller in pH 7.4 than in pH 5.0.

Table 1 gathers the comparison of analytical parameters obtained for the calibration graphs (Figure S2 in Supplementary Materials) reflecting the determination of 4-amino-TEMPO with the use of fluorescence spectroscopy (the measurement of relative fluorescence enhancement of 6,7-dihydroxycoumarin as a function of the concentration of 4-amino-TEMPO within the range 0–500 nM) and UV-Vis spectroscopy (the measurement of absorbance of 4-amino-TEMPO at 375 nm as a function of the concentration of 4-amino-TEMPO within the range 0–50 mM), respectively. Within the investigated ranges of quantitation, strictly linear relationships were observed—as is evidenced by the values of the coefficients of determination (R^2^) close to unity. Both methods are characterized with high accuracy and precision (CV <3%); however the great superiority of fluorescence spectroscopy over spectrophotometry for determination of the studied nitroxide radical lies in the considerably lower value of limit of detection (LOD)—approximately five orders of magnitude. Furthermore, the proposed spectrofluorimetric method based on the use of dihydroxy-substituted coumarins enables the determination of 4-amino-TEMPO at concentrations significantly lower than these reported previously for other nitroxides and EPR spectroscopy [22,42,43].

The measurements of fluorescence lifetimes of 6,7-dihydroxycoumarin alone (0.09 ns) as well as in the presence of 4-amino-TEMPO and NaOH (2.26 and 2.34 ns, respectively) revealed, with high probability, that esculetin undergoes the same reaction under the action of the nitroxide and hydroxide, since the values of lifetimes in the presence of 4-amino-TEMPO or NaOH are within the range of experimental error. As 6,7-dihydroxycoumarin exhibits weak acidic properties due to having phenolic moiety, it can naturally react with sodium hydroxide. Such a route of the reaction should be considered in case of reaction with 4-amino-TEMPO as well.

**Figure 5:**
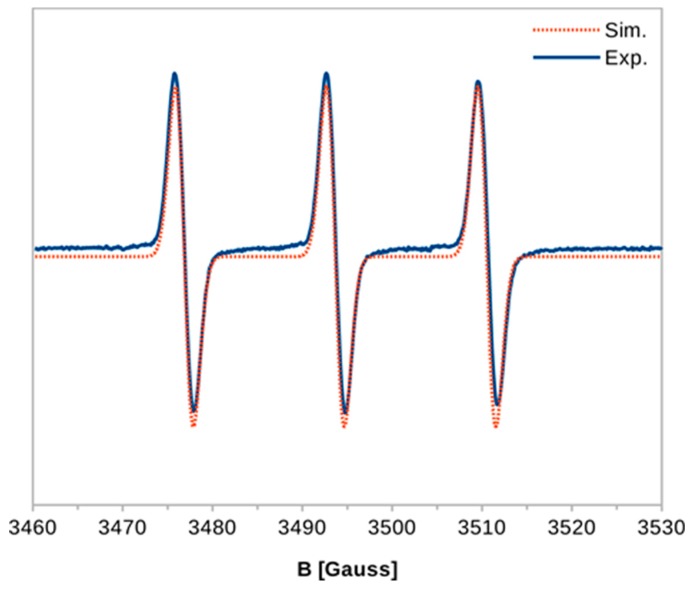
Experimental and simulated EPR spectrum of 4-amino-TEMPO (g_iso_ = 2.00543 and a_iso_(^14^N) = 16.81 G).

The EPR investigation was conducted to confirm that proton transfer occurs from the molecule of 6,7-dihydroxycoumarin to 4-amino-TEMPO radical as well as to reject the possibility of hydrogen atom transfer, which may occur between a phenolic compound and a radical [44]. The EPR spectrum of 4 amino-TEMPO was recorded (Figure 5) and its simulation revealed a set of parameters characteristic for a nitroxide radical in polar environment, namely g_iso_ = 2.00543 and a_iso_(^14^N) = 16.81 G [45,46,47]. If the hydrogen atom transfer from diprotonated (H_2_Q) or monoprotonated (HQ^–^) form of 6,7-dihydroxycoumarin occurred, then a semiquinone radical would be generated in the following process:

H_2_Q + 4-amino-TEMPO → HQ^•^ + 4-amino-TEMPO-H

HQ^–^ + 4-amino-TEMPO → Q^–•^ + 4-amino-TEMPO-H

Semiquinone radicals can be detected in EPR experiments [48,49,50,51], but the EPR spectrum recorded for 4-amino-TEMPO in the presence of 6,7-dihydroxycoumarin (100 µM) did not reveal the formation of semiquinone radical, hence excluding the hydrogen atom transfer in the investigated system. In fact, the EPR spectrum of 4-amino-TEMPO recorded in the presence of 6,7-dihydroxycoumarin was identical to that recorded without esculetin (Figure 5). The addition of 6,7-dihydroxycoumarin did not exert any noticeable effect on the two EPR parameters: g_iso_ = 2.00542 and a_iso_(^14^N) = 16.83 G. Hence, the possibility that the nitroxide moiety >N-O^•^ is the proton acceptor can be excluded. In nitroxides this group is the spin density carrier and its protonation would have a profound effect on the EPR spectrum of 4-amino-TEMPO [52]. This indicates that the reaction between 6,7-dihydroxycoumarin and 4-amino-TEMPO is based on the proton transfer from a hydroxyl group of esculetin onto the amino group of the radical. The protonation of the amino group, which is distant from the paramagnetic centre, should bring about marginal to none change in the spin density and thus no alteration of the g_iso_ and a_iso_(^14^N) parameters for 4-amino-TEMPO should be observed, as in the performed EPR experiment. To confirm this hypothesis the EPR spectrum of 4-amino-TEMPO was recorded in 2-mM solution of HClO_4_, that is, in the conditions enforcing protonation of the amino group but excluding the possibility of the hydrogen atom transfer due to the lack of potential hydrogen atom donor. Again, the g_iso_ and a_iso_(^14^N) parameters derived from the simulation were nearly identical to their counterparts determined for 4-amino-TEMPO in water, g_iso_ = 2.00542 and a_iso_(^14^N) = 16.84 G.

The results obtained from the EPR measurements have subsequently been verified by the investigation of acid-base properties of the reactants. The values of acid dissociation constants (p*K_a_* = −log*K_a_*) obtained from potentiometric titration data are: p*K_a_* = 8.67 (±0.05) for 4-amino-TEMPO and p*K_a_*_1_ = 7.72 (±0.05) and p*K_a_*_2_ = 11.56 (±0.07) for 6,7-dihydroxycoumarin. Consequently, 6,7-dihydroxycoumarin acts as a weak monoprotic acid, whereas 4-amino-TEMPO behaves like a weak base. However, in strongly alkaline solutions, the dissociation of a proton from the second hydroxyl group of 6,7-dihydroxycoumarin is also possible. This confirms, in the system under study, that the proton transfer from the -OH group of 6,7-dihydroxycoumarin to the -NH_2_ group of 4-amino-TEMPO takes place. There is one additional argument supporting protonation of the amino group. It was shown that the p*K_b_* values for *N*-oxides, which are structurally like nitroxide radicals, are close to 10 [53] and therefore the -NH_2_ group should be considered as a significantly stronger proton acceptor in comparison with the >N-O^•^ moiety.

**Figure 6:**
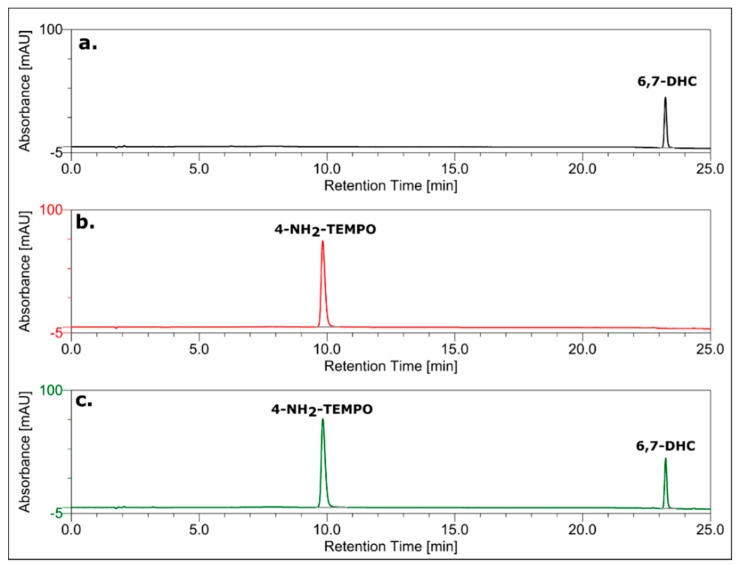
RP-HPLC chromatograms of: (**a**) 0.2 mM 6,7-dihydroxycoumarin; (**b**) 0.2 mM 4-amino-TEMPO; (**c**) the mixture of 0.2 mM 6,7-dihydroxycoumarin and 0.2 mM 4-amino-TEMPO. The chromatograms were recorded at 260 nm.

**Figure 7:**
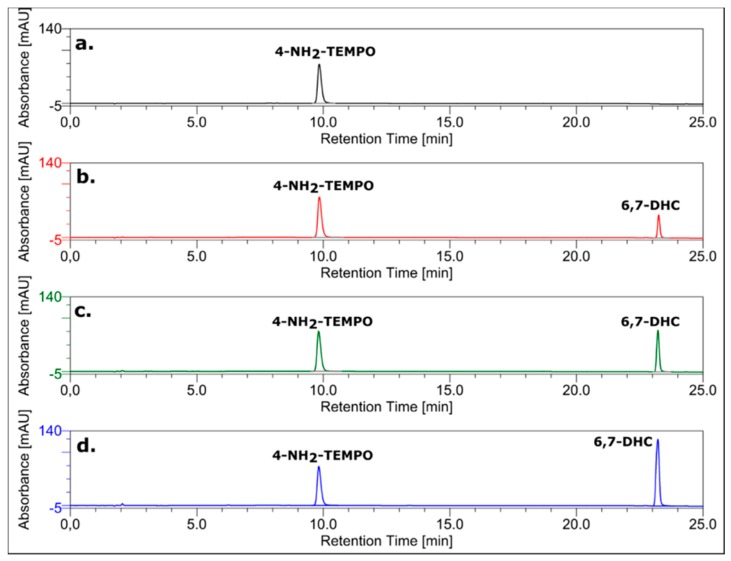
RP-HPLC chromatograms of: (**a**) 0.2 mM 4-amino-TEMPO; (**b**) the mixture of 0.2 mM 4-amino-TEMPO and 0.2 mM 6,7-dihydroxycoumarin; (**c**) the mixture of 0.2 mM 4-amino-TEMPO and 0.4 mM 6,7-dihydroxycoumarin; (**d**) the mixture of 0.2 mM 4-amino-TEMPO and 0.8 mM 6,7-dihydroxycoumarin. The chromatograms were recorded at 260 nm.

The final confirmation of the mechanism based solely on the proton transfer from the hydroxyl group of 6,7-dihydroxycoumarin to the amino group of 4-amino-TEMPO comes from high-performance liquid chromatography experiments. As it can be observed from Figure 6, there is no additional covalent product of the reaction between 6,7-dixydroxycoumarin and 4-amino-TEMPO. Furthermore, the concentration of the nitroxide is not changed when treated with different concentrations of the coumarin (Figure 7). It finally proves that the possibility of a reaction of the amine group with the coumarin ring (or a product of its oxidation) with no doubt can be excluded.

### 2.2. Biological Studies

**Figure 8:**
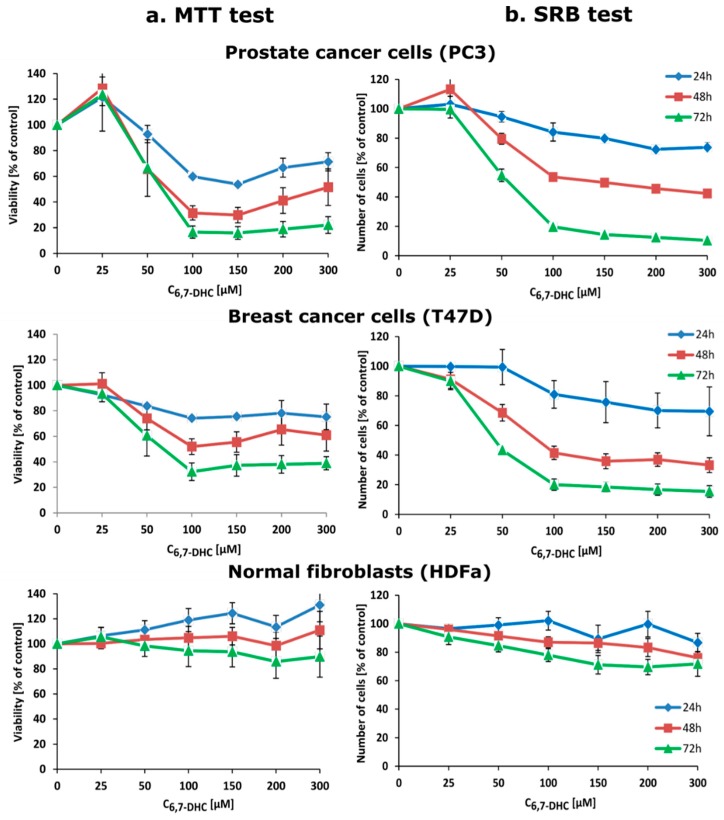
Selective decrease in (**a**) viability and (**b**) number of cancer cells but not normal fibroblasts under the action of 6,7-dihydroxycoumarin, measured by MTT and SRB tests, respectively. The viability and number of cells are shown as mean value ± standard error.

**Figure 9:**
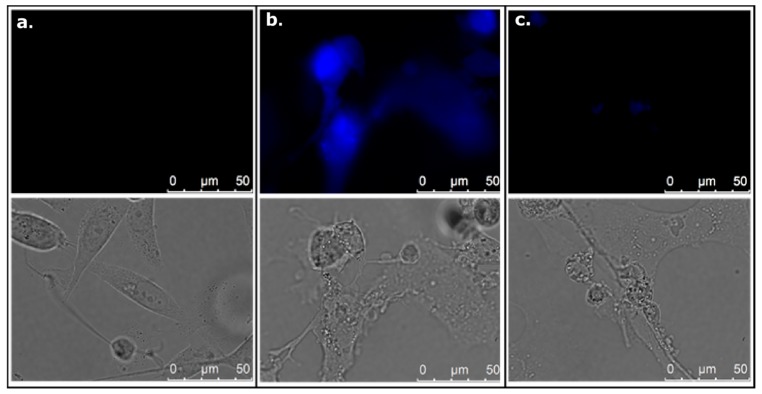
Impact of cellular pH on 6,7-dihydroxycoumarin fluorescence. (**a**) Prostate cancer cells treated with a vehicle (control) and incubated in pH 7.4; (**b**) prostate cancer cells treated with 0.2 mM 6,7-dihydroxycoumarin and incubated in pH 7.4; (**c**) prostate cancer cells treated with 0.2 mM 6,7-dihydroxycoumarin and incubated in pH 6.2 (the use of 5 µM nigericin). Cells were analysed in differential interference contrast (lower panel) and in fluorescence (upper panel).

To introduce a method for detection and determination of 4-amino-TEMPO under biological conditions with the use of 6,7-dihydroxycoumarin we have performed additional experiments on prostate cancer cells (PC3 cell line), breast cancer cells (T47D cell line), and normal skin fibroblasts (HDFa cell line). Firstly, all types of the studied cells were treated with 25–300 µM esculetin for 24, 48, and 72 h. 6,7-Dihydroxycoumarin itself significantly and selectively decreases viability (measured by the MTT test) as well as the number (measured by the SRB test) of cancer cells but not fibroblasts, it can be observed in Figure 8. Furthermore, esculetin affects morphology of cancer cells but not normal fibroblasts (Figure S3 in Supplementary Materials). This clearly demonstrates the anticancer properties of 6,7-dihydroxycoumarin. Interestingly, 6,7-dihydroxycoumarin exhibits strong fluorescence when accumulated in cytoplasm and nuclei of both prostate and breast cancer cells, while no fluorescence is observed in fibroblasts (Figure S4 in Supplementary Materials). There are some possible explanations for such an observation: (1) normal skin fibroblasts are able to remove 6,7-dihydroxycoumarin effectively; (2) 6,7-dihydroxycoumarin does not localize in normal skin fibroblasts due to different parameters of cell membrane or cytosol, i.e., pH (in comparison with cancer cells); and (3) 6,7-dihydroxycoumarin is effectively metabolized in cancer cells due to relatively high oxidative stress status caused by an overproduction of different reactive oxygen and nitrogen species when compared to normal cells. Even though all these possibilities are reasonable, firstly we have decided to check if the difference in fluorescence emission of 6,7-dihydroxycoumarin in various types of cells is associated with the change in intracellular pH, which could be potentially caused by the addition of 4-amino-TEMPO. However, the incubation of prostate cancer cells, breast cancer cells, and normal skin fibroblasts with 4-amino-TEMPO and treated previously with 6,7-dihydroxycoumarin caused no increase in its intracellular fluorescence intensity. We assumed that the nitroxide does not penetrate the cell or penetrates the cell without affecting any change in its intracellular pH. This prompted us to perform an additional experiment in which prostate cancer cells were treated separately with 200 µM 6,7-dihydroxycoumarin for 48 h and then incubated in a dark for 2 min: (1) in pure HBSS buffer (pH 7.4) and (2) with 5 µM nigericin in HBSS buffer (pH adjusted to 6.2). The obtained results unequivocally revealed that in pH 6.2 esculetin exhibits no fluorescence, while in pH 7.4 it emits strongly (Figure 9). It is in a good agreement with a chemical experiment, since greater pH favours the deprotonation of 6,7-dihydroxycoumarin and consequently is responsible for the higher observed fluorescence emission (and, at the same time, lower intensity enhancement caused by the addition of the nitroxide). Furthermore, since the most significant decreases in viability and number of cancer cells go hand in hand with the greatest fluorescence emission intensities values, taking into account the fact that, due to a significant pH gradient between extra- and intracellular space of cancer cells, the vast majority of the molecules of 6,7-dihydroxycoumarin inside these cells are ionized. It can thus be assumed that deprotonated form of esculetin is the one that exhibits the strongest anticancer properties. On the other hand, additional experiments in that field—which are currently underway in our laboratory—are required.

## 3. Materials and Methods

### 3.1. Materials

The reagents, namely 4-amino-TEMPO (purity 97%) and a series of 19 coumarin derivatives (purity 98%), i.e., 6,7-dihydroxycoumarin; 7,8-dihydroxy-6-methoxycoumarin; 5,7-dihydroxy-4-methylcoumarin; 7,8-dihydroxy-4-phenylcoumarin; 7-hydroxy-4-methylcoumarin; 3-[4-(bromomethyl)phenyl]-7-(diethylamino)coumarin; 3-acetylcoumarin; 7-hydroxycoumarin; 7,8-dihydroxy-4-methylcoumarin; 7-diethylamino-4-methylcoumarin; 7-ethoxy-4-methylcoumarin; 7-methoxycoumarin; 5,7-dihydroxy-4-phenylcoumarin; 5-methoxypsoralene; 3-chlorocoumarin; 7,8-dihydroxycoumarin; 4,7-dihydroxycoumarin; 6-methoxy-7-hydroxycoumarin; and 7-hydroxy-4-methyl-3-coumarinylacetic acid, were purchased from Sigma Aldrich (Poland) and used as obtained. The doubly distilled water with a conductivity not exceeding 0.18 μS/cm was used as a solvent. The stock solutions of 4-amino-TEMPO and the coumarins studied were prepared by dissolving an appropriate amount of the substance in water (with a sonication) and kept in the dark at 4 °C. To avoid a self-quenching or inner filter effect, the solutions of all coumarins used for stationary fluorescence experiments were prepared at a fixed concentration (10 μM). The concentration of the stock solution of 4-amino-TEMPO was equal to 0.10 M.

### 3.2. UV-Vis Spectroscopy

UV-Vis absorption spectra of the studied coumarins were recorded in water, at a concentration 0.10 mM, in the absence and presence of 4-amino-TEMPO at concentrations up to 0.50 mM, using a Perkin Elmer Lambda 650 UV-Vis spectrophotometer. The spectra were registered at 25 °C.

### 3.3. Steady-State Fluorescence Spectroscopy

The fluorescence emission spectra of the studied coumarins (10 µM) were registered in aqueous solutions with the use of a Cary Eclipse Varian spectrofluorimeter (excitation and emission slits—5 nm; PMT detector voltage—600 V) in the absence and presence of 4-amino-TEMPO at concentrations up to 0.10 mM. Excitation wavelength was individually chosen for each coumarin based on its UV-Vis absorption spectrum. The fluorescence intensity values were always measured at the maximum of the emission, immediately (1–2 s) after the addition of 4-amino-TEMPO and mixing the solution (spectrofluorimeter equipped with magnetic stirrer), which caused (if any) distinct and rapid changes by leaps and bounds. The values of excitation and emission wavelengths are gathered in Table 2. The emission spectra as well as all fluorescence intensity measurements were performed at 25 °C. The possible absorption of light by 4-amino-TEMPO at the excitation and emission wavelengths of coumarins has been considered during data analysis. The extent of the inner filter effect was estimated based on the following equation:

$$F_{\mathrm{corr}}=F_{\mathrm{obs}}\times 10^{\frac{A_{\mathrm{ex}}+A_{\mathrm{em}}}{2}}$$

where F_corr_ and F_obs_ are the corrected and observed fluorescence intensities, respectively, whereas A_ex_ and A_em_ are the sum of the absorbance of the appropriate coumarin and the nitroxide radical at the excitation and emission wavelength, respectively [54].

### 3.4. Time-Resolved Fluorescence Spectroscopy

Time-resolved fluorescence measurements were performed with a FluoTime 300 high performance fluorescence lifetime spectrometer (PicoQuant) at 20 °C. The excitation source was pulsed LED of the PLS series (λ_ex_ = 340 nm). There were measured (in water as a solvent) the fluorescence lifetimes of: (1) 0.10 mM 6,7-dihydroxycoumarin; (2) 0.10 mM 6,7-dihydroxycoumarin in the presence of 0.50 mM 4-amino-TEMPO and (3) 0.10 mM 6,7-dihydroxycoumarin in the presence of 0.50 mM NaOH (λ_em_ = 354 nm).

### 3.5. Electron Paramagnetic Resonance

Electron paramagnetic resonance (EPR) spectra were recorded at room temperature, using a Bruker Elexsys E500 spectrometer equipped with an NMR teslameter and a frequency counter, operating at a microwave power of 20 mW and a modulation amplitude of 1 G. Smaller microwave powers (1–20 mW) and modulation amplitudes (0.1–1 G) were also tested. In all EPR experiments water was used as a solvent and the concentration of 4-amino-TEMPO was 75 μM. The simulations of the experimental spectra were performed using the “EPR of S > ½” computer program written by dr. A. Ozarowski (National High Magnetic Field Laboratory, Florida State University, Tallahassee, FL, USA).

### 3.6. Potentiometric Titration

Potentiometric titrations were performed in 30 mL of thermostated (25 ± 0.10 °C) cells using Cerko Lab System microtitration unit fitted with 5-mL Hamilton’s syringe and a pH-combined electrode (Schott-BlueLine 16 pH type). The electrode was calibrated according to IUPAC recommendations [55]. All details for the measuring devices and the experimental setup were described previously [56]. All the solutions were prepared immediately before the measurements. The compositions of the titrand solutions used in the experiments were as follows: (1) 1 mM 4-amino-TEMPO and 2 mM HClO_4_ and (2) 1 mM 6,7-dihydroxycoumarin and 2 mM HClO_4_. The solutions were potentiometrically titrated with a standardized NaOH solution (24.4 mM) at pH ranging from 3.0 to 12.0. The acid dissociation constants of 4-amino-TEMPO and 6,7-dihydroxycoumarin were determined using CVEQUID program [57] by minimization of the differences between the theoretical model and the experimental data, according to Gauss-Newton-Marquardt for nonlinear equations [58].

### 3.7. HPLC Analysis

To monitor the interaction between 6,7-dihydroxycoumarin and 4-amino-TEMPO, reversed-phase chromatography (RP HPLC) was applied using a HPLC Dionex UltiMate 3000 System equipped with UV-DAD detector. The chromatograms were recorded at 260 nm for monitoring the effluents. The Wakopak reverse-phase C18 column (4.6 mm × 150 mm; particle size = 5 µm) was used. Separations were performed using linear gradient from 0 to 50% of phase B in 25 min (A: 0.1 M ammonium acetate, pH 5.5; B: 80% AcN in water; flow rate = 1 mL/min).

### 3.8. Cell Culture

Monolayers of prostate cancer cell line (PC3) and breast cancer cell line (T47D) were maintained in F12K or RPMI medium, respectively, supplemented with 9% (PC3) or 10% (T47D) foetal bovine serum and antibiotics, as described in [59] and [60], respectively. Normal human dermal fibroblasts (HDFa) were maintained in DMEM medium with high glucose and sodium pyruvate, supplemented with antibiotics and 10% heat-inactivated foetal bovine serum. Each cell line was maintained at 37 °C in a humidified atmosphere with 5% CO_2_. 6,7-Dihydroxycoumarin was prepared in dimethyl sulfoxide (DMSO) as a series of stock solutions with different concentration. Control cells were treated with an equal amount of a pure solvent (DMSO). For the analysis of 6,7-dihydroxycoumarin intracellular fluorescence, cells were washed and observed in Dulbecco’s Phosphate Buffered Saline (PBS) or Hank’s Balanced Salt Solution (HBSS) supplemented with 10 mM HEPES with pH adjusted to 7.4.

### 3.9. Cell Viability Assay

Cells viability was determined by the MTT test (in which mitochondrial metabolic activity of cells is used as an indirect measure of cells viability) as described previously [61]. 4 × 10^3^ cells per well were seeded in a 96-well plate and allowed to attach overnight. Next day, medium was replaced with fresh one supplemented with equal amount of a pure vehicle (DMSO) or 25, 50, 100, 200 or 300 µM 6,7-dihydroxycoumarin for 24–72 h. Then, the MTT solution was added to a final concentration of 1 mg/mL and cells were incubated for next 3 h. After this time, medium was removed, and formazan crystals were dissolved in 100 µL of DMSO. Absorbance in wells was measured at 570 nm (with reference wavelength 620 nm) in Victor3 microplate reader. The viability of control was taken as 100%. Data were obtained from three independent experiments, each treatment condition assayed in duplicate.

### 3.10. Indirect Analysis of Cell Number

Indirect analysis of cell number was performed by the SRB test (in which the amount of protein is used as an indirect measure of cells number) as described previously [62]. Briefly, cells were seeded at a density of 4 × 10^3^/well of 96-well plate. Twenty-four hours later, cells were treated with equal amount of a pure vehicle (DMSO) or 25, 50, 100, 200 or 300 µM 6,7-dihydroxycoumarin for 24–72 h. After this time, medium was removed and 100 µL per well of 20% trichloroacetic acid was added in 4 °C. Then, after 1 h, wells were washed with water, stained with 0.4% sulforhodamine B solution in 1% acetic acid for 15 min, and washed again (three times) with 1% acetic acid. Afterwards, 150 µL per well of 10 mM Tris base (pH 10.5) was added. The absorbance was measured at 570 nm with a reference filter of 660 nm in a Victor3 microplate reader. The absorbance of control was taken as 100%. Data were obtained from three independent experiments, each treatment condition assayed in duplicate.

### 3.11. Analysis of Cell Morphology in Phase Contrast Microscopy

PC3, T47D and HDFa cells were seeded at a density of 4 × 10^3^/well of 96-well plate and were treated the next day with a pure DMSO (control) or 50, 150, and 300 µM 6,7-dihydroxycoumarin for 72 h. Cell morphology was assessed with the use of inverted microscope with phase contrast at a total magnification of 100×.

### 3.12. Analysis of Intracellular 6,7-Dihydroxycoumarin Localization Using Fluorescence Microscope

PC3, T47D, or HDFa cells were seeded at a density of 7.5 × 10^4^/well on coverslips placed in 12-well plate. 24 h later cells were treated with vehicle (DMSO; control) or 100 µM 6,7-dihydroxycoumarin for 72 h. Then, coverslips were washed with PBS and coated with CitiFluor non-hardening mounting solution. Cells were analysed immediately in differential interference contrast and in fluorescence using appropriate filter set (λ_ex_ = 340–380 nm; λ_em_ = 450–490 nm) and constant acquisition parameters, which were chosen so that no autofluorescence of control (DMSO-treated) cells was observed. 100× immersion oil objective was applied. The experiment was performed in at least three independent replicates.

### 3.13. Impact of Cellular pH on 6,7-Dihydroxycoumarin Fluorescence

Modulation of intracellular pH was performed as described previously [63]. Prostate cancer cells (PC3) were seeded on coverslips in 35 mm dishes, allowed to grow overnight and treated with a vehicle (control) or 6,7-dihydroxycoumarin (200 µM) for 48 h. Then, control cells were incubated for 2 min in the dark in pure HBSS buffer (pH 7.4), whereas cells treated with 6,7-dihydroxycoumarin were either treated the same or incubated in the dark in 5 µM nigericin in HBSS buffer with pH adjusted to 6.2. Cells were analysed immediately in differential interference contrast and in fluorescence using appropriate filter set (λ_ex_ = 340-380 nm; λ_em_ = 450–490 nm) and constant acquisition parameters, which were chosen so that no autofluorescence of control (DMSO-treated) cells was observed. Then, 100× immersion oil objective was applied. The experiment was performed in two independent replicates.

## 4. Conclusions

The above data present the development and evaluation of dihydroxycoumarins for sensitive and selective detection and determination of 4-amino-TEMPO in aqueous solutions. The use of a series of complementary techniques enabled us to prove that the mechanism of the interaction between 6,7-dihydroxycoumarin and 4-amino-TEMPO is based on a proton transfer from the molecule of esculetin to the amino group of the nitroxide radical. Under the action of 4-amino-TEMPO, 6,7-dihydroxycoumarin undergoes deprotonation, which is accompanied by a significant increase in fluorescence intensity as well as fluorescence lifetime. Esculetin responds to 4-amino-TEMPO quickly and shows an up to 40-fold linear fluorescence enhancement for 0.1 mM nitroxide with a limit of detection equal to approximately 16.7 nM. The studies performed in different pH revealed that when pH of the solution increases, the observed fluorescence intensity enhancement under the action of 4-amino-TEMPO decreases (better sensing properties of dihydroxycoumarins towards the nitroxide in lower pH). The method is characterized by an easy procedure of sample preparation and a very short time of analysis. Biological studies revealed significant anticancer properties of 6,7-dihydroxycoumarin and showed that the fluorescence emission of esculetin in cancer cells is closely related to intracellular pH. Since 4-amino-TEMPO is used as a spin probe for the detection of various radicals and damage caused by these species, its detection and determination under physiological conditions might help us to understand the mechanism of the oxidative stress.

## Figures and Tables

**Table 1:** Analytical characteristic of calibration graph reflecting the relationship between the relative fluorescence enhancement of 6,7-dihydroxycoumarin and the concentration of 4-amino-TEMPO compared with spectrophotometric method (absorbance of 4-amino-TEMPO measured at λ_max_ = 375 nm).

	Method	Fluorescence Spectroscopy (for 6,7-Dihydroxycoumarin)	UV-Vis Spectroscopy
Validation Parameter			
Range of quantitation (M)		5 × 10^−8^–5 × 10^−5^	2.5 × 10^−3^–5 × 10^−2^
Coefficient of determination (R^2^)		0.9912	0.9986
Coefficient of variation CV (%)		2.56	2.87
Accuracy (%)		92–106	92–105
Limit of detection (LOD) (M)		1.67 × 10^−8^	0.833 × 10^−3^
Limit of quantitation (LOQ) (M)		5 × 10^−8^	2.5 × 10^−3^

**Table 2:** Excitation and emission wavelengths of the studied coumarins.

Coumarin Derivative	λ_ex_ (nm)	λ_em_ (nm)
6,7-dihydroxycoumarin	350	467
7,8-dihydroxy-6-methoxycoumarin	380	517
5,7-dihydroxy-4-methylcoumarin	330	457
7,8-dihydroxy-4-phenylcoumarin	340	510
5,7-dihydroxy-4-phenylcoumarin	360	460
7,8-dihydroxy-4-methylcoumarin	330	528
3-[4-(bromomethyl)phenyl]-7-(diethylamino)coumarin	410	492
3-acetylcoumarin	370	458
3-chlorocoumarin	330	442
7-hydroxycoumarin	360	456
7-hydroxy-4-methylcoumarin	340	451
7-diethylamino-4-methylcoumarin	400	470
7-ethoxy-4-methylcoumarin	330	387
7-methoxycoumarin	340	394
5-methoxypsoralene	320	455
7-hydroxy-4-methyl-3-coumarinylacetic acid	350	456
7,8-dihydroxycoumarin	330	450
4,7-dihydroxycoumarin	310	418
6-methoxy-7-hydroxycoumarin	350	462

## Supplementary Materials

Supplementary materials can be found at https://www.mdpi.com/1422-0067/20/15/3802/s1.
