# Supplementary material for: Dihydroxy-Substituted Coumarins as Fluorescent Probes for Nanomolar-Level Detection of the 4-Amino-TEMPO Spin Label
Source: Int J Mol Sci. 2019 Aug 3;20(15):3802. doi: 10.3390/ijms20153802 (PMC6696051; doi:10.3390/ijms20153802)
Supplement: Supplementary file 1 [file ijms-20-03802-s001.pdf]

(Electronic Supplementary Material)

Dihydroxy-Substituted Coumarins as Fluorescent Probes for Nanomolar Level Detection of 4-Amino-TEMPO Spin Label

Krzysztof Żamojć[a]*, Magdalena Zdrowowicz[a], Aleksandra Hać[b], Maciej Witwicki[c], Paweł B. Rudnicki-Velasquez[a], Dariusz Wyrzykowski[a], Wiesław Wiczk[a], and Lech Chmurzyński[a]

[a] Faculty of Chemistry, University of Gdańsk, Wita Stwosza 63, 80-308 Gdańsk, Poland;
[b] Faculty of Biology, University of Gdańsk, Wita Stwosza 59, 80-308 Gdańsk, Poland;
[c] Faculty of Chemistry, University of Wrocław, F. Joliot-Curie 14, 50-383 Wrocław, Poland

magdalena.zdrowowicz@ug.edu.pl (M.Z.); aleksandra.wiczk@biol.ug.edu.pl (A.H.); maciej.witwicki@chem.uni.wroc.pl (M.W.); rudnicki.pb@gmail.com (P.R.-V.); dariusz.wyrzykowski@ug.edu.pl (D.W.); wieslaw.wiczk@ug.edu.pl (W.W.); lech.chmurzynski@ug.edu.pl (L.C.)

*Correspondence: krzysztof.zamojc@ug.edu.pl (K.Ż.); Tel.: +48 58 523 50 57

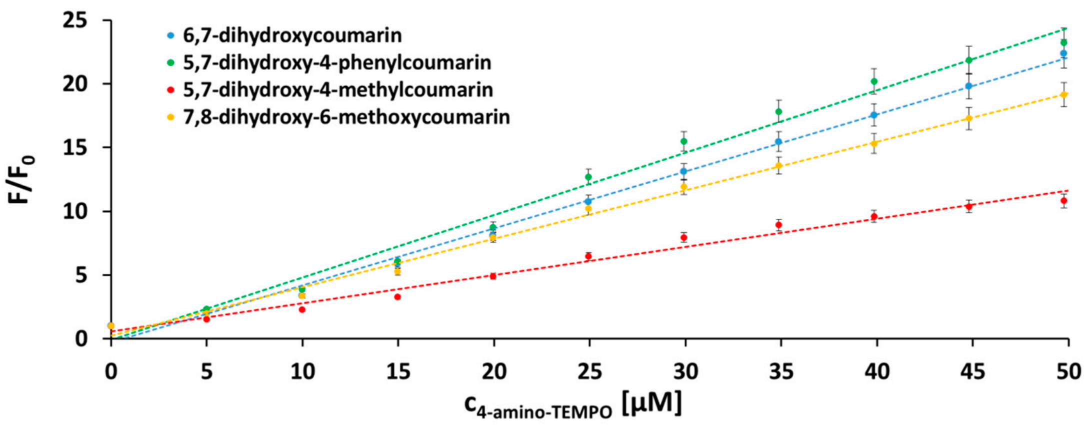

Figure S1. 4-Amino-TEMPO concentration-dependent fluorescence intensity enhancement for 6,7-dihydroxycoumarin, 5,7-dihydroxy-4-methylcoumarin, 5,7-dihydroxy-4-phenylcoumarin and 7,8-dihydroxy-6-methoxycoumarin. The concentration of each coumarin is 10 μM. Each data represents an average of 3 independent experiments, while fluorescence intensity values are an average of 15 experiments. For details see the Experimental section.

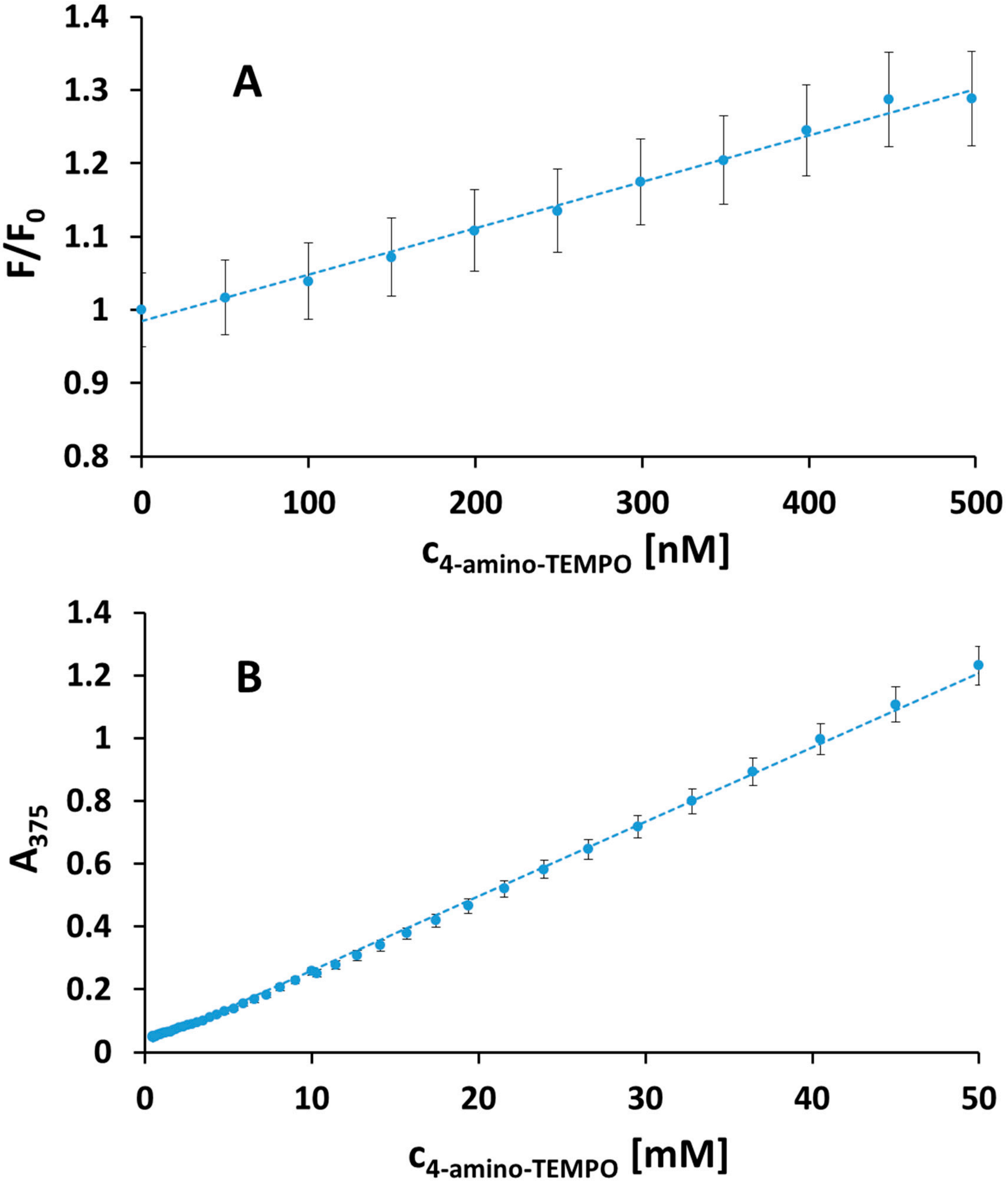

Figure S2. Calibration graphs reflecting the relationship between: (A) the relative fluorescence enhancement of 6,7-dihydroxycoumarin and the concentration of 4-amino-TEMPO within the range 0 – 500 nM; and (B) absorbance of 4-amino-TEMPO measured at 375 nm and the concentration of 4-amino-TEMPO within the range 0 – 50 mM. Each data represents an average of 3 independent experiments, while fluorescence intensity values are an average of 15 experiments. For details see Table 1.

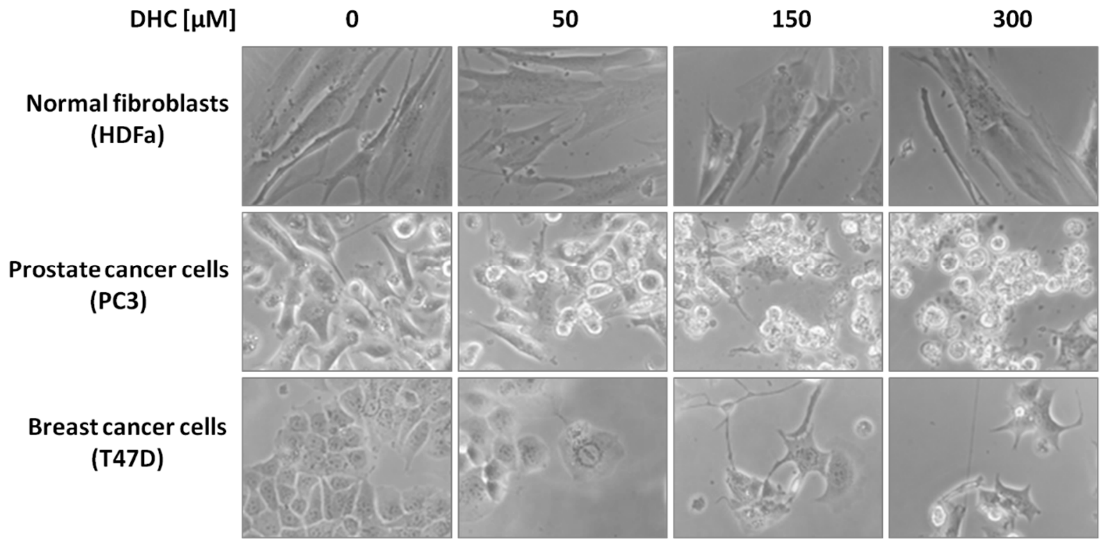

Figure S3. Impact of 6,7-dihydroxycoumarin on morphology of cancer cells in comparison with normal fibroblasts (all treated with indicated concentrations of 6,7-dihydroxycoumarin for 72 hours).

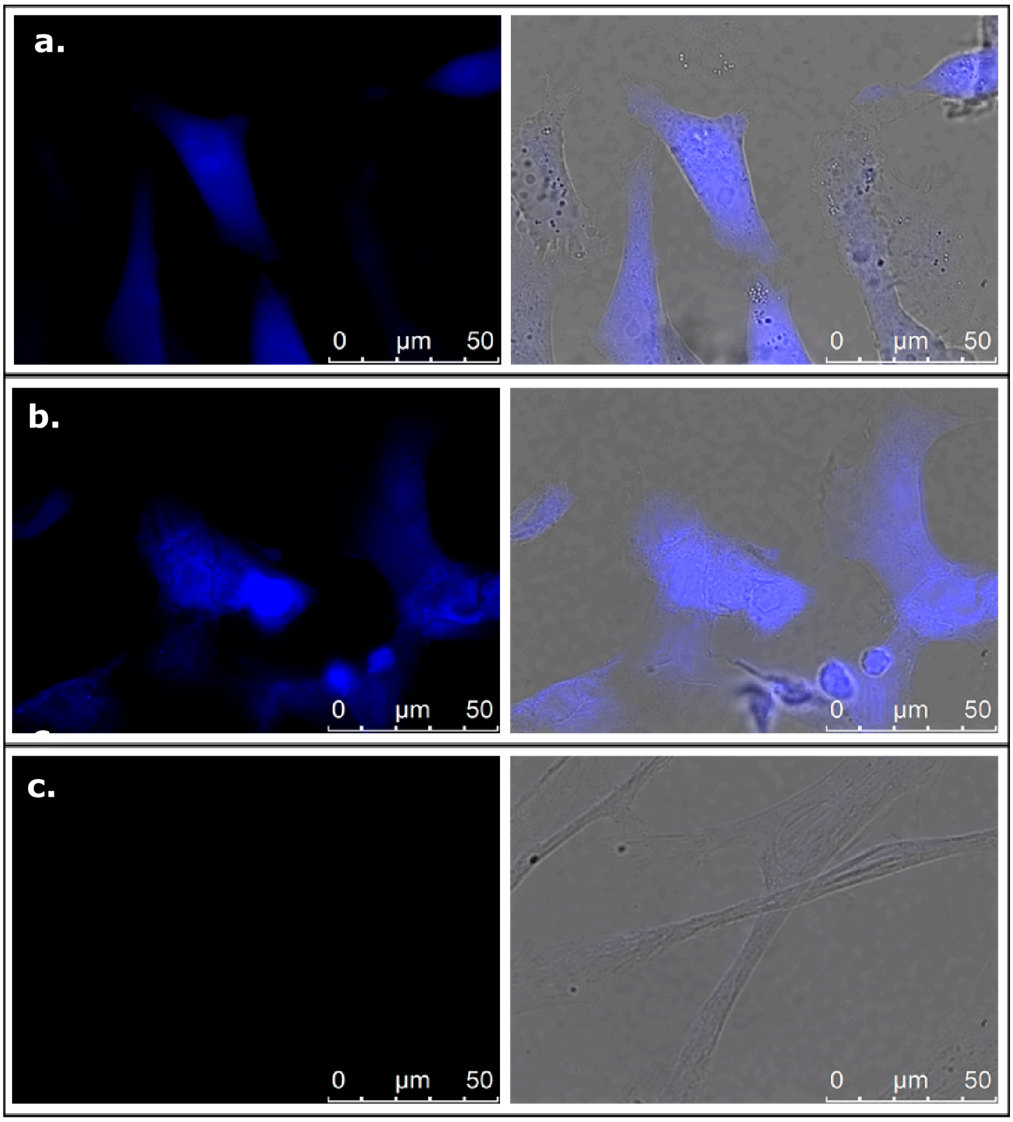

Figure S4. Analysis of intracellular 6,7-dihydroxycoumarin localization. 6,7-Dihydroxycoumarin accumulates in cytoplasm and nucleus of cancer cells but not in normal fibroblasts. (a) Prostate cancer cells, (b) breast cancer cells, and (c) normal fibroblasts analyzed in fluorescence (left panel) and differential interference contrast (merged with fluorescence; right panel).
